# Inflammatory Response Genes’ Polymorphism Associated with Risk of Rheumatic Heart Disease

**DOI:** 10.3390/jpm14070753

**Published:** 2024-07-15

**Authors:** Anna Sinitskaya, Maria Khutornaya, Oksana Hryachkova, Maxim Asanov, Alyona Poddubnyak, Anastasia Ponasenko, Maxim Sinitsky

**Affiliations:** 1 Laboratory of Genome Medicine, Research Institute for Complex Issues of Cardiovascular Diseases, 650002 Kemerovo, Russia; 2 State Healthcare Institution City Hospital No. 7, 300012 Tula, Russia

**Keywords:** rheumatic heart disease, native heart valves, bacterial invasion, infection, inflammation, inflammatory response, genetic polymorphism, cytokines, interleukins, toll-like receptors

## Abstract

Rheumatic heart disease (RHD) caused by group A streptococcus infection is one of the most important reasons of cardiovascular morbidity and mortality in low- and middle-income countries. Aberrant host immune response modulated by polymorphisms in inflammatory response genes plays an important role in RHD pathogenesis. This study aimed to determine risk-associated polymorphic variants in inflammatory response genes in Caucasian RHD patients. A total of 251 Caucasian RHD patients and 300 healthy donors were recruited for this study, and 27 polymorphic sites in 12 genes (*TLR1*, *TLR2*, *TLR4*, *TLR6*, *IL1B*, *IL6R*, *IL6*, *IL10*, *IL12RB1*, *IL12B*, *TNF* and *CRP*) were analyzed using allele-specific PCR. It was demonstrated that the polymorphic variants rs1800871 and rs1800872 in the IL10 gene, rs 1130864, rs3093077 and rs1205 in the CRP gene, rs375947 in the IL12RB1 gene, rs 5743551 and rs5743611 in the TLR1 gene, and rs3775073 in the TLR6 gene can modify RHD risk in a gender- and age-dependent manner. The obtained results can be used to determine the personalized risk of RHD in healthy donors during medical examination or screening, as well as to develop appropriate early prevention strategies targeting RHD in the risk groups.

## 1. Introduction

Rheumatic heart disease (RHD) resulting from the dysfunction of native heart valves remains one of the most important reasons for cardiovascular morbidity and mortality mainly in low- and middle-income countries [1]. According to the statistics, RHD is the main cause of heart failure in children and young adults [2]. Improving RHD diagnostics (e.g., early echocardiography-based screening) is challenging to its epidemiology due to the identification of silent cases of this pathology and is very important as a secondary prevention strategy [3,4].

RHD is caused by an abnormal immune response consisting of humoral and cellular components of *Streptococcus pyogenes*, mainly after a throat infection [5,6]. RHD pathogenesis is based on the triad of the rheumatogenic group A streptococcal strain, a genetically susceptible host, and an aberrant host immune response [7]. Innate immune response mediates via toll-like receptors (TLRs) which primarily identify various pathogens by recognizing pathogen-associated molecular patterns (PAMPs) and damage-associated molecular patterns (DAMPs) [8,9]. Single nucleotide polymorphisms (SNPs) in the *TLR* genes modulate TLR activity, signal transduction from the activated receptor into cells [10] and, finally, the adequate response of human organism to infection [11,12,13]. It should be noted that the currently available data on the contribution of genetic polymorphism in the *TLR* genes to RHD pathogenesis are limited and contradictory [14,15,16,17].

RHD is associated with a chronic aseptic inflammation leading to the fibrosis of native heart valves and, finally, to their dysfunction [18]. A number of cytokines and chemokines mediating an inflammatory response (IL-1β, IL-6, IL-8/CXCL8, IL-10, IL-18, TNFα, etc.) play the central role in RHD pathogenesis [19,20,21,22,23]. It is known that an inflammatory response is genetically determined [24], so it can be suggested that genetic polymorphism in cytokine genes is associated with RHD risk and severity in a population-dependent manner [19,25,26]. Genome-wide association studies (GWAS) allow for the identification of novel genetic variants associated with RHD pathogenesis [27,28] that confirm the significant contribution of the genetic component to the RHD pathogenesis.

This study aimed to determine risk-associated polymorphic variants in inflammatory response genes in Caucasian RHD patients.

## 2. Materials and Methods

### 2.1. Group Description

A total of 251 Caucasian patients aged 29 to 77 years (mean age of 57 years), hospitalized at the Research Institute for Complex Issues of Cardiovascular Diseases (Kemerovo, Russia) from 2016 to 2022 due to RHD, were recruited for this study. RHD was established on the basis of clinical and anamnestic data; all patients underwent mitral valve-replacement under artificial circulation. Patients with instrumentally confirmed mitral stenosis or regurgitation accompanied by infective endocarditis, absence of rheumatic anamnesis, or presence of severe concomitant pathology were excluded from the study. The full clinical characteristic of RHD patients is presented in Table 1. A total of 300 healthy donors without any signs of cardiovascular, autoimmune, mental, infectious diseases, cancer, or exacerbation of chronic diseases aged 21 to 80 years (mean age of 53 years) who underwent a routine medical examination at the Research Institute for Complex Issues of Cardiovascular Diseases (Kemerovo, Russia) from 2016 to 2019 were recruited into the control group.

The study design was approved by the Institutional Review Board of the Research Institute for Complex Issues of Cardiovascular Diseases (Kemerovo, Russia). All patients recruited in the present study provided written informed consent to participate in the examination. This study complies with the Declaration of Helsinki (ethical principles for medical research involving human participants, amended in 2000) and Good Clinical Practice guidelines.

### 2.2. Molecular Genetic Testing

Genomic DNA was extracted from whole blood collected in vacuum tubes containing K3EDTA using the routine phenol-chloroform extraction method. SNPs were selected according to the following criteria: (i) location within genes involved in the inflammatory response; (ii) minor allele frequency > 5% in Caucasian population; (iii) functional consequences and related studies on their role in RHD pathogenesis. Based on these criteria, 27 SNPs in 12 genes were selected: *TLR1* (rs5743551 and rs5743611), *TLR2* (rs3804099 and rs5743708), *TLR4* (rs4986790 and rs4986791), *TLR6* (rs3775073 and rs5743810), *IL1B* (rs1143634 and rs16944), *IL6R* (rs2228145 and rs2229238), *IL6* (rs1800796, rs1554606 and rs2069827), *IL10* (rs1800871, rs1800872 and rs1800896), *IL12RB1* (rs375947), *IL12B* (rs3212227), *TNF* (rs1799964, rs361525 and rs1800629) and *CRP* (rs3093077, rs1800947, rs1130864 and rs1205) (Table 2).

Selected SNPs were analyzed by allele-specific real-time PCR with fluorescently labeled TaqMan primers (Applied Biosystems, Waltham, MA, USA) using ViiA 7 Real-Time PCR System (Applied Biosystems, Waltham, MA, USA). Per each analyzed sample, 10 μL of reaction mixture containing 1.25 μL of TaqMan primers, 1 mM of dNTP (Life Technologies, Carlsbad, CA, USA), 1U of TaqDNA polymerase (Life Technologies, Carlsbad, CA, USA) and 100 ng of DNA template was prepared. The amplification was performed as follows: 10 min at 95 °С, 15 s at 95 °С and 60 s at 60 °С (40 cycles). Repeated genotyping of 10% of samples was performed to control the PCR quality.

### 2.3. Statistical Analysis

Statistical analysis was performed using the Prism 7 (v.7.04) software package (GraphPad Software, Boston, MA, USA) and the SNPStats web tool (https://www.snpstats.net/tutorial.htm, accessed on 23 May 2024). The D’Agostino–Pearson normality test was applied to verify the compliance of the obtained data with normal distribution. Quantitative data were processed using the Yates’ chi-square test. To identify risk/protective SNPs, the odds ratio (OR) and 95% confidence interval (CI) were calculated. The most likely inheritance model for each specific gene polymorphism was determined using Akaike’s information criterion (AIC). The differences were considered statistically significant at *p* < 0.05.

## 3. Results

Resulting from molecular genetic testing, the A/A genotype in the *IL10* (rs1800871) gene (OR = 2.54, 95% CI = 1.24–5.18, *p* = 0.0084), the T/T genotype in the *IL10* (rs1800872) gene (OR = 2.33, 95% CI = 1.16–4.69, *p* = 0.014) and the T/T genotype in the *CRP* (rs1130864) gene (OR = 1.76, 95% CI = 1.09–2.83, *p* = 0.02) were associated with an increased RHD risk in the recessive inheritance model. The polymorphic variant rs3093077 in the *CRP* gene was characterized by a protective effect in the log-additive inheritance model (OR = 0.53, 95% CI = 0.30–0.94, *p* = 0.026) (Table 3). 

After stratification by gender, we found that the A/A genotype in the IL10 (rs1800871) gene (OR = 3.69, 95% CI = 1.45–9.42, *p* = 0.003), the T/T genotype in the IL10 (rs1800872) gene (OR = 3.90, 95% CI = 1.54–9.91, *p* = 0.0017), the C/C genotype in the TLR1 (rs5743551) gene (OR = 2.37, 95% CI = 1.08–5.21, *p* = 0.027) and the G/G genotype in the TLR1 (rs5743611) gene (OR = 4.07, 95% CI = 1.31–12.61, *p* = 0.0076) were associated with an increased RHD risk in the recessive inheritance model in female patients. The polymorphic variant rs3093077 in the CRP gene was characterized by a protective effect in the log-additive inheritance model (OR = 0.45, 95% CI = 0.22–0.92, *p* = 0.022) (Table 4). In male patients we found no statistically significant associations between genetic polymorphism and RHD risk. 

The polymorphic variants rs1800871 and rs1800872 of the *IL10* gene were also associated with an increased RHD risk in middle-aged patients (age ≤ 60 years). In addition, the carriers of the T/T genotype in the *CRP* (rs1205) gene (OR = 2.35, 95% CI = 1.28–4.32, *p* = 0.0061) in the recessive inheritance model were characterized by an increased RHD risk, and the carriers of the A/G genotype in the *IL12RB1* (rs375947) gene had a decreased risk of this pathology (OR = 0.57, 95% CI = 0.36–0.93, *p* = 0.022) (Table 5).

In the group of elderly (age > 60 years) patients, only two risk genotypes were identified—the C/G genotype in the TLR1 (rs5743611) gene and the T/C genotype in the TLR6 gene, both in the overdominant inheritance model (OR = 1.98, 95% CI = 1.07–3.67, *p* = 0.026 and OR = 2.02, 95% CI = 1.18–3.48, *p* = 0.01, respectively) (Table 6).

## 4. Discussion

The cardiovascular risk in RHD patients is partly associated with RHD-associated systemic inflammation [29]. Despite the fact that the molecular mechanisms underlying RHD pathophysiology are still not fully understood, evidence suggests that inflammatory response plays a critical role in the pathogenesis of RHD [30,31,32]. Briefly, bacterial invasion by group A streptococcus (GAS) induces a delayed autoimmune response, resulting in acute rheumatic fever (ARF), while a recurrent or continuing autoimmune inflammatory response against self-antigens triggers autoimmune-driven RHD [33,34]. Immune response mediated through a number of cytokines, chemokines, adhesion receptors, proteases, autoantibodies and toll-like receptors allows us to consider these molecules as key to the RHD pathogenesis and maintenance of cardiovascular homeostasis. 

It is known that SNPs in the regulatory regions of cytokine genes modulate cytokine release and activity in response to infectious, allergic, autoimmune and malignant diseases [35,36]. Resulting from our study, the homozygous genotype of the *IL10* gene in the recessive inheritance model is associated with the more than two-fold increased RHD risk in the most studied models (patients without stratification by gender or age, female patients and middle-aged patients). The *IL10* gene encoding an important immunoregulatory molecule, interleukin-10 (IL-10) [37], was mainly produced by T-regulatory type 1 (Tr1) cells [38,39]. IL-10 is an anti-inflammatory cytokine inhibiting IL-12, IFN-γ and MHC II expression, regulating immune cells, tolerance to self-antigens and antigen presentation [40,41,42,43]. A number of studies demonstrated the overexpression of IL-10 in ARF and RHD patients [42,44,45]. IL-10 can be involved in RHD pathogenesis due to its ability to induce antibody production. It was shown that an increased IL-10 level is associated with an elevated production of autoantibody in patients with autoimmune diseases [46,47] via altering the regulation of B-cell bcl-2 expression [48]. IL-10 also promotes the development of the Th2 type cytokine pattern via the inhibition of IL-12 and IFN-γ [49,50,51,52] and induces recruitment and cytotoxic activity of CD8+ cells infiltrated into heart valves [53]. Infiltrating CD8+ T-cells might contribute to increased cell cytotoxicity into heart valves and their damage. So, it can be suggested that IL-10 overexpression is associated with an increased risk of heart valve damage [19].

The role of IL-10 on bacterial clearance or bacterial persistence [54,55] was also demonstrated. In the case of intracellular bacterial infections, the IL-10 overexpression is associated with the host’s immune suppression and bacterial persistence. Although *Streptococcus pyogenes* is an extracellular pathogen, it can invade the cytoplasm of the phagocytic cells [56,57], so a high level of IL-10 can lead to the its persistence, which is critical to RHD progression.

It should be noted than the role of genetic polymorphism in the *IL10* gene in RHD pathogenesis is still poorly investigated, and the available results are still contradictory. In some studies, no significant association between the *IL10* gene polymorphism and RHD risk were reported [58,59,60]. On the other hand, a number of studies demonstrated an increased RHD risk in carriers of the heterozygous genotype G/A of polymorphic variant –1082 A/G in the Egyptian population [61,62] and the homozygous genotype A/A in the recessive inheritance model in the Saudi Arabian population [63]. 

C-reactive protein (CRP) is a polypeptide molecule playing an important role in innate immunity, immunoglobulins receptor-binding and complement activation. CRP is a protein of acute systemic inflammation widely used as a prime marker of inflammation [64]. It is known that RHD patients are characterized by an increased serum blood level of CRP due to inflammation accompanying this disease [65,66,67,68], but the role of genetic polymorphism in the *CRP* gene in RHD risk has still not been investigated. Resulting from our study, the polymorphic variants in the *CRP* gene are associated with both increased and decreased RHD risk in the Caucasian population, but this depends on the studied polymorphic variants. CRP binds to plasma membranes of damaged cells and some types of bacteria [69] characterized by overexpression of lysophospholipid and followed by the activation of complement [70]. It can be suggested that genetic polymorphism in the *CRP* gene modulates ligand-recognition and -binding by CRP, which may contribute to a range of metabolic, scavenging and host-defense functions. 

Interleukin 12 (IL-12) is a pro-inflammatory cytokine produced in response to bacterial invasion [71] and which plays a key role in the development of Th1 cells [72]. Pathogen-associated molecular patterns (lipopolysaccharide, teichoic acid, peptidoglycan and bacterial CpG DNA) induce IL-12 secretion. The biological role of IL-12 is to promote innate and adaptive immunity. It is involved in the differentiation of naive CD4+ T-cells to Th1 cells, activation of natural killer cells producing IFN-γ and other type-1 cytokines, protection CD4+ T-cells from antigen-induced apoptotic death and promotion of T-cell trafficking and migration into sites of immune response [73]. The differential secretion of IL-12 in response to bacterial invasion depends on the differences in the regulation of the IL12 gene, patterns of TLR expression and cross-regulation between the different subsets, involving cytokines such as IL-10 and type I IFN [74]. IL-12 signals via interleukin 12 receptors IL12Rβ1 and IL12Rβ2 [75]. It was shown that the serum blood level of IL-12 was significantly higher in RHD patients [76], but the role of genetic polymorphism in the genes encoding IL-12 and its receptors in the RHD pathogenesis is still unknown. Resulting from our research, the polymorphic variant rs375947 in the IL12RB1 gene is associated with the decreased RHD risk in middle-aged patients from the Caucasian population.

The recognition of pathogenic microorganisms by innate immune systems is mainly ensured by TLRs—the members of pattern-recognition receptors. TLRs can recognize and bind such pathogen-associated molecular patterns as lipopolysaccharide, peptidoglycan, lipopeptides, bacterial DNA and flagellin followed by the initiating of acute inflammatory response against pathogens [8,77]. TLRs trigger phagocytosis activation, the direct killing of the pathogens and the initiation of adaptive immune response through co-stimulatory molecule expression on dendritic cells [78]. It was shown that the polymorphisms in the *TLR* genes are associated with the risk of bacterial infections [79,80,81,82,83,84,85] and streptococcal-associated ARF [86]. It should be noted that the role of polymorphisms in the *TLR* genes in RHD risk is still poorly investigated, and the available results remain contradictory. In some studies, no significant association with RHD risk was reported [14,15]; and some researchers demonstrated the associations of polymorphic variants in the *TLR* genes and RHD risk [16,17]. Moreover, it was shown that the role of TLRs in autoimmune inflammatory diseases was characterized by the dysregulation of the immune system, which finally resulted in the break of tolerance to self-antigen resulting from inappropriate TLR stimulation [87,88]. In the presented research, we discovered for the first time the risk alleles in the *TLR1* and *TLR6* genes in the Caucasian population.

Resulting from our study, the gender- and age-specific associations of immune response gene polymorphisms with the RHD risk were discovered. It is known that gender dimorphism of various biological and physiological functions is caused by differences in gonadal hormone secretion, which can vary in a gender- and age-dependent manner, and this physiological alteration can influence the function of gonadal hormone-sensitive genes [89]. Gonadal hormones can significantly modulate cell signaling pathways and control gene regulation and expression. It was shown that gonadal hormones may modify the immune response via regulating the production of pro- and anti-inflammatory cytokines and TLR expression [90,91,92,93].

It should be noted that this study was limited to analyzing only genetic polymorphism in immune response genes, and the serum blood level of the inflammatory markers (cytokines, CRP, etc.) was not measured. Due to this limitation, the clinical significance of the discovered RHD risk-associated polymorphic variants is debatable.

## 5. Conclusions

Genetic polymorphisms in inflammatory response genes are associated with RHD risk in the Caucasian population in a gender- and age-depended manner. The obtained results can be used to determine the personalized risk of RHD in healthy donors during medical examination or screening, as well as to develop appropriate early prevention strategies targeting RHD in the risk groups. 

## Figures and Tables

**Table 1:** Clinical and demographic characteristics of patients included in the study.

Index	RHD Patients	Control Group
Male, N (%)	65 (25.9)	110 (36.7)
Female, N (%)	186 (74.1)	180 (63.3)
Age, M (Q1; Q3)	57 (29; 77)	53 (21; 80)
Mitral stenosis, N (%)	162 (64.6)	-
Mitral regurgitation, N (%)	89 (35.5)	-
Pulmonary hypertension, N (%)	204 (79.1)	-
Chronic heart failure, N (%)	250 (96.8)	-
NYHA Class I, N (%)	1 (0.4)	-
NYHA Class II, N (%)	27 (10.8)	-
NYHA Class III, N (%)	211 (84.4)	-
NYHA Class IV, N (%)	11 (4.4)	-
Left ventricular ejection fraction, M (Q1; Q3)	60.62 (28; 85)	-
Atrial fibrillation, N (%)	192 (74.1)	-
Hypertensive heart disease, N (%)	143 (55.4)	-
Acute cerebrovascular accident, N (%)	19 (7.4)	-
Type 2 diabetes, N (%)	19 (7.4)	-
Obesity, N (%)	42 (28.9)	-

**Table 2:** Characteristics of the selected polymorphic variants.

Gene	Encoding Protein	Reference SNP Number	Chromosomal Position	Nucleotide Change
*TLR1*	Toll-like Receptor 1	rs5743551	chr4:38806033	T > A, C, G
		rs5743611	chr4:38798593	C > G
*TLR2*	Toll-like Receptor 2	rs3804099	chr4:153703504	T > C
		rs5743708	chr4:153705165	G > A
*TLR4*	Toll-like Receptor 4	rs4986790	chr9:117713024	A > G, T
		rs4986791	chr9:117713324	C > T
*TLR6*	Toll-like Receptor 6	rs3775073	chr4:38828211	T > C, G
		rs5743810	chr4:38828729	A > C, G, T
*IL1B*	Interleukin 1 Beta	rs1143634	chr2:112832813	G > A
		rs16944	2:112837290	A > G
*IL6R*	Interleukin 6 Receptor	rs2228145	chr1:154454494	A > C, T
		rs2229238	chr1:154465420	T > A, C
*IL6*	Interleukin 6	rs1800796	chr7:22726627	G > A, C
		rs1554606	chr7:22729088	T > A, G
		rs2069827	chr7:22725837	G > C, T
*IL10*	Interleukin 10	rs1800871	chr1:206773289	A > G
		rs1800872	chr1:206773062	T > G
		rs1800896	chr1:206773552	T > C
*IL12RB1*	Interleukin 12 Receptor Subunit Beta 1	rs375947	chr19:18069641	A > G
*IL12B*	Interleukin 12 Subunit Beta	rs3212227	chr5:159315942	T > G
*TNF*	Tumor Necrosis Factor	rs1799964	chr6:31574531	T > C
		rs361525	chr6:31575324	G > A
		rs1800629	chr6:31575254	G > A
*CRP*	C-Reactive Protein	rs3093077	chr1:159709846	A > C, G, T
		rs1800947	chr1:159713648	C > A, G, T
		rs1130864	chr1:159713301	G > A
		rs1205	chr1:159712443	C > T

**Table 3:** Statistically significant associations of genetic variants in the immune response genes with the risk of rheumatic heart disease.

Gene	Model	Genotype	RHD Patients, *N* (%)	Control Group, *N* (%)	OR (95% CI)	*p*-Value	AIC
*IL10* rs1800871	Codominant	G/G	148 (59%)	183 (61%)	1.00	0.029	758.4
		A/G	79 (31.5%)	105 (35%)	0.93 (0.65–1.34)		
		A/A	24 (9.6%)	12 (4%)	2.47 (1.20–5.11)		
	Dominant	G/G	148 (59%)	183 (61%)	1.00	0.63	763.2
		A/G-A/A	103 (41%)	117 (39%)	1.09 (0.77–1.53)		
	Recessive	G/G-A/G	227 (90.4%)	288 (96%)	1.00	0.0084	756.5
		A/A	24 (9.6%)	12 (4%)	**2.54 (1.24–5.18)**		
	Overdominant	G/G-A/A	172 (68.5%)	195 (65%)	1.00	0.38	762.7
		A/G	79 (31.5%)	105 (35%)	0.85 (0.60–1.22)		
	Log-additive	-	-	-	1.22 (0.93–1.60)	0.15	761.4
*IL10* rs1800872	Codominant	G/G	150 (59.8%)	185 (61.7%)	1.00	0.047	759.4
		T/G	77 (30.7%)	102 (34%)	0.93 (0.65–1.34)		
		T/T	24 (9.6%)	13 (4.3%)	2.28 (1.12–4.62)		
	Dominant	G/G	150 (59.8%)	185 (61.7%)	1.00	0.65	763.3
		T/G-T/T	101 (40.2%)	115 (38.3%)	1.08 (0.77–1.53)		
	Recessive	G/G-T/G	227 (90.4%)	287 (95.7%)	1.00	0.014	757.5
		T/T	24 (9.6%)	13 (4.3%)	**2.33 (1.16–4.69)**		
	Overdominant	G/G-T/T	174 (69.3%)	198 (66%)	1.00	0.41	762.8
		T/G	77 (30.7%)	102 (34%)	0.86 (0.60–1.23)		
	Log-additive	-	-	-	1.20 (0.92–1.58)	0.18	761.7
*CRP* rs1130864	Codominant	C/C	89 (35.5%)	112 (37.3%)	1.00	0.066	760
		C/T	116 (46.2%)	154 (51.3%)	0.95 (0.66–1.37)		
		T/T	46 (18.3%)	34 (11.3%)	1.70 (1.01–2.87)		
	Dominant	C/C	89 (35.5%)	112 (37.3%)	1.00	0.65	763.3
		C/T-T/T	162 (64.5%)	188 (62.7%)	1.08 (0.77–1.54)		
	Recessive	C/C-C/T	205 (81.7%)	266 (88.7%)	1.00	0.02	758.1
		T/T	46 (18.3%)	34 (11.3%)	**1.76 (1.09–2.83)**		
	Overdominant	C/C-T/T	135 (53.8%)	146 (48.7%)	1.00	0.23	762.1
		C/T	116 (46.2%)	154 (51.3%)	0.81 (0.58–1.14)		
	Log-additive	-	-	-	1.21 (0.95–1.55)	0.13	761.2
*CRP* rs3093077	Codominant	C/C	233 (92.8%)	262 (87.3%)	1.00	0.066	760.1
		A/C	18 (7.2%)	37 (12.3%)	0.55 (0.30–0.99)		
		A/A	0 (0%)	1 (0.3%)	0.00 (0.00-NA)		
	Dominant	C/C	233 (92.8%)	262 (87.3%)	1.00	0.031	758.8
		A/C-A/A	18 (7.2%)	38 (12.7%)	0.53 (0.30–0.96)		
	Recessive	C/C-A/C	251 (100%)	299 (99.7%)	1.00	0.27	762.3
		A/A	0 (0%)	1 (0.3%)	0.00 (0.00-NA)		
	Overdominant	C/C-A/A	233 (92.8%)	263 (87.7%)	1.00	0.042	759.3
		A/C	18 (7.2%)	37 (12.3%)	0.55 (0.30–0.99)		
	Log-additive	-	-	-	**0.53 (0.30–0.94)**	0.026	758.5

**Note:** Statistically significant models after applying Akaike’s information criterion (AIC) are highlighted in bold. NA: not applicable.

**Table 4:** Statistically significant associations of genetic variants in the immune response genes with the risk of rheumatic heart disease in female patients.

Gene	Model	Genotype	RHD Patients, *N* (%)	Control Group, *N* (%)	OR (95% CI)	*p*-Value	AIC
*IL10* rs1800871	Codominant	G/G	111 (59.7%)	119 (62.6%)	1.00	0.011	518.2
		A/G	55 (29.6%)	65 (34.2%)	0.91 (0.58–1.41)		
		A/A	20 (10.8%)	6 (3.2%)	3.57 (1.38–9.22)		
	Dominant	G/G	111 (59.7%)	119 (62.6%)	1.00	0.56	524.9
		A/G-A/A	75 (40.3%)	71 (37.4%)	1.13 (0.75–1.71)		
	Recessive	G/G-A/G	166 (89.2%)	184 (96.8%)	1.00	0.003	516.4
		A/A	20 (10.8%)	6 (3.2%)	**3.69 (1.45–9.42)**		
	Overdominant	G/G-A/A	131 (70.4%)	125 (65.8%)	1.00	0.33	524.3
		A/G	55 (29.6%)	65 (34.2%)	0.81 (0.52–1.25)		
	Log-additive	-	-	-	1.32 (0.95–1.83)	0.1	522.5
*IL10* rs1800872	Codominant	G/G	112 (60.2%)	120 (63.2%)	1.00	0.0064	517.1
		T/G	53 (28.5%)	64 (33.7%)	0.89 (0.57–1.39)		
		T/T	21 (11.3%)	6 (3.2%)	3.75 (1.46–9.63)		
	Dominant	G/G	112 (60.2%)	120 (63.2%)	1.00	0.56	524.9
		T/G-T/T	74 (39.8%)	70 (36.8%)	1.13 (0.75–1.72)		
	Recessive	G/G-T/G	165 (88.7%)	184 (96.8%)	1.00	0.0017	515.4
		T/T	21 (11.3%)	6 (3.2%)	**3.90 (1.54–9.91)**		
	Overdominant	G/G-T/T	133 (71.5%)	126 (66.3%)	1.00	0.28	524
		T/G	53 (28.5%)	64 (33.7%)	0.78 (0.51–1.22)		
	Log-additive	-	-	-	1.33 (0.96–1.84)	0.086	522.3
*CRP* rs3093077	Codominant	C/C	174 (93.5%)	165 (86.8%)	1.00	0.059	521.5
		A/C	12 (6.5%)	24 (12.6%)	0.47 (0.23–0.98)		
		A/A	0 (0%)	1 (0.5%)	0.00 (0.00-NA)		
	Dominant	C/C	174 (93.5%)	165 (86.8%)	1.00	0.027	520.3
		A/C-A/A	12 (6.5%)	25 (13.2%)	0.46 (0.22–0.94)		
	Recessive	C/C-A/C	186 (100%)	189 (99.5%)	1.00	0.24	523.8
		A/A	0 (0%)	1 (0.5%)	0.00 (0.00-NA)		
	Overdominant	C/C-A/A	174 (93.5%)	166 (87.4%)	1.00	0.04	521
		A/C	12 (6.5%)	24 (12.6%)	0.48 (0.23–0.98)		
	Log-additive	-	-	-	**0.45 (0.22–0.92)**	0.022	520
*TLR1* rs5743551	Codominant	T/T	99 (57.6%)	105 (55.3%)	1.00	0.039	500.5
		T/C	53 (30.8%)	75 (39.5%)	0.75 (0.48–1.17)		
		C/C	20 (11.6%)	10 (5.3%)	2.12 (0.95–4.76)		
	Dominant	T/T	99 (57.6%)	105 (55.3%)	1.00	0.66	504.7
		T/C-C/C	73 (42.4%)	85 (44.7%)	0.91 (0.60–1.38)		
	Recessive	T/T-T/C	152 (88.4%)	180 (94.7%)	1.00	0.027	500.1
		C/C	20 (11.6%)	10 (5.3%)	**2.37 (1.08–5.21)**		
	Overdominant	T/T-C/C	119 (69.2%)	115 (60.5%)	1.00	0.085	502
		T/C	53 (30.8%)	75 (39.5%)	0.68 (0.44–1.06)		
	Log-additive	-	-	-	1.10 (0.80–1.52)	0.55	504.6
*TLR1* rs5743611	Codominant	C/C	100 (57.5%)	122 (64.2%)	1.00	0.024	502.4
		C/G	60 (34.5%)	64 (33.7%)	1.14 (0.74–1.78)		
		G/G	14 (8.1%)	4 (2.1%)	4.27 (1.36–13.38)		
	Dominant	C/C	100 (57.5%)	122 (64.2%)	1.00	0.19	506.2
		C/G-G/G	74 (42.5%)	68 (35.8%)	1.33 (0.87–2.03)		
	Recessive	C/C-C/G	160 (92%)	186 (97.9%)	1.00	0.0076	500.8
		G/G	14 (8.1%)	4 (2.1%)	**4.07 (1.31–12.61)**		
	Overdominant	C/C-G/G	114 (65.5%)	126 (66.3%)	1.00	0.87	507.9
		C/G	60 (34.5%)	64 (33.7%)	1.04 (0.67–1.60)		
	Log-additive	-	-	-	1.45 (1.02–2.06)	0.04	503.7

**Note:** Statistically significant models after applying Akaike’s information criterion (AIC) are highlighted in bold. NA: not applicable.

**Table 5:** Statistically significant associations of genetic variants in the immune response genes with the risk of rheumatic heart disease in middle-aged patients (age ≤ 60 years).

Gene	Model	Genotype	RHD Patients, *N* (%)	Control Group, *N* (%)	OR (95% CI)	*p*-Value	AIC
*IL10* rs1800871	Codominant	G/G	66 (55.5%)	126 (60.9%)	1.00	0.048	427.8
		A/G	39 (32.8%)	72 (34.8%)	1.03 (0.63–1.69)		
		A/A	14 (11.8%)	9 (4.3%)	2.97 (1.22–7.22)		
	Dominant	G/G	66 (55.5%)	126 (60.9%)	1.00	0.34	431
		A/G-A/A	53 (44.5%)	81 (39.1%)	1.25 (0.79–1.97)		
	Recessive	G/G-A/G	105 (88.2%)	198 (95.7%)	1.00	0.014	425.8
		A/A	14 (11.8%)	9 (4.3%)	**2.93 (1.23–7.00)**		
	Overdominant	G/G-A/A	80 (67.2%)	135 (65.2%)	1.00	0.71	431.7
		A/G	39 (32.8%)	72 (34.8%)	0.91 (0.57–1.47)		
	Log-additive	-	-	-	1.38 (0.97–1.97)	0.076	428.7
*IL10* rs1800872	Codominant	G/G	66 (55.5%)	127 (61.4%)	1.00	0.045	427.7
		T/G	38 (31.9%)	70 (33.8%)	1.04 (0.64–1.71)		
		T/T	15 (12.6%)	10 (4.8%)	2.89 (1.23–6.78)		
	Dominant	G/G	66 (55.5%)	127 (61.4%)	1.00	0.3	430.8
		T/G-T/T	53 (44.5%)	80 (38.6%)	1.27 (0.81–2.01)		
	Recessive	G/G-T/G	104 (87.4%)	197 (95.2%)	1.00	0.013	425.7
		T/T	15 (12.6%)	10 (4.8%)	**2.84 (1.23–6.55)**		
	Overdominant	G/G-T/T	81 (68.1%)	137 (66.2%)	1.00	0.73	431.8
		T/G	38 (31.9%)	70 (33.8%)	0.92 (0.57–1.49)		
	Log-additive	-	-	-	1.40 (0.98–1.98)	0.063	428.4
*IL12RB1* rs375947	Codominant	A/A	59 (50.9%)	96 (46.4%)	1.00	0.023	420.2
		A/G	36 (31%)	91 (44%)	0.64 (0.39–1.07)		
		G/G	21 (18.1%)	20 (9.7%)	1.71 (0.85–3.42)		
	Dominant	A/A	59 (50.9%)	96 (46.4%)	1.00	0.44	425.2
		A/G-G/G	57 (49.1%)	111 (53.6%)	0.84 (0.53–1.32)		
	Recessive	A/A-A/G	95 (81.9%)	187 (90.3%)	1.00	0.032	421.2
		G/G	21 (18.1%)	20 (9.7%)	2.07 (1.07–4.00)		
	Overdominant	A/A-G/G	80 (69%)	116 (56%)	1.00	0.022	420.5
		A/G	36 (31%)	91 (44%)	**0.57 (0.36–0.93)**		
	Log-additive	-	-	-	1.09 (0.78–1.50)	0.62	425.5
*CRP* rs1205	Codominant	C/C	40 (33.6%)	82 (39.6%)	1.00	0.023	426.3
		C/T	52 (43.7%)	102 (49.3%)	1.05 (0.63–1.73)		
		T/T	27 (22.7%)	23 (11.1%)	2.41 (1.23–4.71)		
	Dominant	C/C	40 (33.6%)	82 (39.6%)	1.00	0.28	430.7
		C/T-T/T	79 (66.4%)	125 (60.4%)	1.30 (0.81–2.08)		
	Recessive	C/C-C/T	92 (77.3%)	184 (88.9%)	1.00	0.0061	424.4
		T/T	27 (22.7%)	23 (11.1%)	**2.35 (1.28–4.32)**		
	Overdominant	C/C-T/T	67 (56.3%)	105 (50.7%)	1.00	0.33	430.9
		C/T	52 (43.7%)	102 (49.3%)	0.80 (0.51–1.26)		
	Log-additive	-	-	-	1.44 (1.04–2.00)	0.027	427

**Note:** Statistically significant models after applying Akaike’s information criterion (AIC) are highlighted in bold.

**Table 6:** Statistically significant associations of genetic variants in the immune response genes with the risk of rheumatic heart disease in elderly patients (age > 60 years).

Gene	Model	Genotype	RHD Patients, *N* (%)	Control Group, *N* (%)	OR (95% CI)	*p*-Value	AIC
*TLR1* rs5743611	Codominant	C/C	72 (57.6%)	66 (71%)	1.00	0.081	298.5
		C/G	44 (35.2%)	20 (21.5%)	2.02 (1.08–3.77)		
		G/G	9 (7.2%)	7 (7.5%)	1.18 (0.42–3.34)		
	Dominant	C/C	72 (57.6%)	66 (71%)	1.00	0.042	297.3
		C/G-G/G	53 (42.4%)	27 (29%)	1.80 (1.02–3.19)		
	Recessive	C/C-C/G	116 (92.8%)	86 (92.5%)	1.00	0.93	301.5
		G/G	9 (7.2%)	7 (7.5%)	0.95 (0.34–2.66)		
	Overdominant	C/C-G/G	81 (64.8%)	73 (78.5%)	1.00	0.026	296.6
		C/G	44 (35.2%)	20 (21.5%)	**1.98 (1.07–3.67)**		
	Log-additive	-	-	-	1.41 (0.90–2.19)	0.13	299.2
*TLR6* rs3775073	Codominant	T/T	33 (25.2%)	35 (37.6%)	1.00	0.033	303.2
		T/C	72 (55%)	35 (37.6%)	2.18 (1.17–4.07)		
		C/C	26 (19.9%)	23 (24.7%)	1.20 (0.57–2.50)		
	Dominant	T/T	33 (25.2%)	35 (37.6%)	1.00	0.047	304.1
		T/C-C/C	98 (74.8%)	58 (62.4%)	1.79 (1.01–3.19)		
	Recessive	T/T-T/C	105 (80.2%)	70 (75.3%)	1.00	0.39	307.3
		C/C	26 (19.9%)	23 (24.7%)	0.75 (0.40–1.43)		
	Overdominant	T/T-C/C	59 (45%)	58 (62.4%)	1.00	0.01	301.5
		T/C	72 (55%)	35 (37.6%)	**2.02 (1.18–3.48)**		
	Log-additive	-	-	-	1.16 (0.80–1.68)	0.44	307.4

**Note:** Statistically significant models after applying Akaike’s information criterion (AIC) are highlighted in bold.

## Data Availability

The data presented in this study are available on request from the corresponding author. The data are not publicly available due to privacy statements.
